# Older People’s Use and Nonuse of the Internet in Sweden

**DOI:** 10.3390/ijerph17239050

**Published:** 2020-12-04

**Authors:** Peter Anderberg, Lisa Skär, Linda Abrahamsson, Johan Sanmartin Berglund

**Affiliations:** 1 Department of Health, Blekinge Institute of Technology, SE-371 79 Karlskrona, Sweden; lisa.skar@bth.se (L.S.); linda.abrahamsson@bth.se (L.A.); johan.sanmartin.berglund@bth.se (J.S.B.); 2 School of Health Sciences, University of Skövde, SE-541 28 Skövde, Sweden

**Keywords:** gerontechnology, aging, internet, usage

## Abstract

The use of the internet has considerably increased over recent years, and the importance of internet use has also grown as services have gone online. Sweden is largely an information society like other countries with high reported use amongst European countries. In line with digitalization development, society is also changing, and many activities and services today take place on the internet. This development could potentially lead to those older persons who do not use the internet or do not follow the development of services on the internet finding it difficult to take part in information and activities that no longer occur in the physical world. This has led to a digital divide between groups, where the older generations (60+), in particular, have been affected. In a large study of Sweden’s adult population in 2019, 95 percent of the overall population was said to be internet users, and the corresponding number for users over 66 years of age was 84%. This study shows that the numbers reported about older peoples’ internet use, most likely, are vastly overestimated and that real use is significantly lower, especially among the oldest age groups. We report that 62.4% of the study subjects are internet users and that this number most likely also is an overestimation. When looking at nonresponders to the questionnaire, we find that they display characteristics generally attributed to non-use, such as lower education, lower household economy, and lower cognitive functioning.

## 1. Introduction

The use of the internet has considerably increased over recent years, and the importance of internet use has also grown as services have gone online. Sweden is largely an information society like other countries with high reported use amongst European countries [1]. In line with digitalization development, society is also changing, and many activities and services today take place on the internet [2]. This leads to people who do not use the internet or do not follow the development of services on the internet, finding it difficult to take part in information and activities that no longer take place physically. This has led to a digital divide between groups, where the older generations, in particular, have been affected [3].

About 98 percent of Sweden’s adult population was reported to have access to the internet in 2019 [1,4], and 95 percent claimed to be internet users and felt involved in the information society as described in the yearly report on the internet habits of the Swedish population: “The Swedes and the Internet 2019” (Svenskarna och Internet 2019(SoI)) [4]. The report also describes differences between those who are digitally active and those who are not, which are decreasing. However, it is estimated that there are 1.1 million (eleven percent) Swedes who do not use the internet and a common factor is age. The reported increase of internet users in the older age group is attributed to the fact that those who retired ten-to-fifteen years ago had become accustomed to internet users in their workplaces. Today they have reached the age of 76 or older and continue to be internet users. In the second-oldest age group, 66–75 years, 93% are reported as internet users, which is on par with the population as a whole. In total, internet use in the 66+ age group is reported to be 84 percent. Compared to internet use among Europeans aged over 50 years in general, only 49 percent use the internet [5,6].

Variations in internet use are of concern because the inability to use the internet can negatively affect older peoples’ daily life and health, as access to services and information can be difficult without internet usage [7,8]. At the same time, there is a need for the digitization of health care services for older people, and they have to be arranged to meet the needs and cope with the financing of welfare when the older population is increasing [9,10]. Information and communication technologies (ICT) offer the potential to improve efficiency in health care systems [11]. ICT and eHealth can be seen as facilitators to access health services, and it can enable autonomy among older people [2,10], and, as older people are expected to live longer in their homes, eHealth services can support their independence. The use of the internet is also shown to promote wellbeing [12], support active aging [13], and be a possible support in maintaining cognitive function [14,15].

As technology is continually changing, flexible skills and technological self-efficacy are needed to use the devices [16]. Additionally, memory and selective attention [17] and cognitive functioning tend to decline with age, which may affect the ability to use devices [18] and commence internet use as well [19]. Thus, it is important to consider cognition when studying internet use. In most studies, there are community-dwelling older adults in the sample, while people with cognitive impairment are often excluded [3].

However, the inability to use the internet can negatively affect older peoples’ daily lives as more and more e-Health services are available online [20]. Therefore, older peoples’ experiences may improve our understanding of their use of the internet to meet expectations and needs.

The aim of the study was to investigate older peoples’ use and non-use of the internet in Sweden.

## 2. Method

### 2.1. Recruitment

Data from the Swedish National Study on Aging and Care (SNAC) was used. SNAC is a longitudinal study conducted in four research centers in Sweden. For this study, data from Karlskrona municipality in Southern Sweden was chosen. The study started in the year 2000, and its cohort contains a subset of the aging population, comprising individuals aged 60 years or older, representing approximately 10 percent of the older adult population in the municipality. Data are collected every 3rd year with questionnaires, interviews, and medical examinations. Every 6th year a new cohort of individuals 60 years old are recruited. The study is designed to collect data about participants’ medical, psychological, and functional statuses, social situation, and care needed [21]. In addition to the core study protocol, 733 participants, all from the sample who were alive in May 2019 and aged 65 years or older, were invited to respond to a questionnaire related to their internet and ICT use in 2019. The response rate was 79.3%. However, 6.7% of the persons could still be included as they were confirmed not to be able to use the internet due to, for example, a relative giving such information or having a diagnosis of severe dementia. The study sample thus consisted of 630 participants, giving an inclusion rate of 85.9%. The questionnaire dropout is presented in Table 1. A total of 103 persons not responding to the questionnaire were excluded from the study.

The Research Ethics Committee approved the study of Lund University (LU 604-00).

### 2.2. Measures

Participants’ internet use was investigated by asking whether they use the internet. Those who had never used the internet or used it before but quit were defined as non-users. Others were defined as internet users. Age was used as both a continuous and a categorized variable with three groups: 65–74 years, 75–84 years, and at least 85 years (the oldest person being 100 years). Sex was used as a binary variable. According to the previous Swedish education system, education was categorized into three groups, relevant for the age groups in this study: low level of education (those who did not finish secondary school), middle level of education (those who finished secondary school), and high level of education (those with some form of higher education). The household economy was measured by asking the participants if they are able to obtain 14,000 SEK within a week to cover an unforeseen expense, with a yes and no answer possible, being interpreted as having a good or poor household economy. The living arrangement was measured by asking if the participant was living alone or living with someone (a partner being the most common person to live with). Cognition functioning was measured using the Swedish translation of the ‘Mini-Mental State Examination’ (MMSE), where a maximum score of 30 means that one has normal cognitive functioning. MMSE was used as a dichotomized variable into poor and normal cognitive functioning using a cut-off point of 27, where 28–30 points were coded as normal functioning [22]. Health state was measured in the questionnaire by asking how well the participant’s health was on a scale from one to five (poor, moderate, good, very good, excellent). In the analyses, the variable was dichotomized so that the first two answers were categorized as poor and the rest as good. Loneliness was categorized into yes or no, depending on the participant’s answers to the question regarding whether they sometimes feel alone or not. The same principle for practical support was used: whether the participants have someone who can help them either at times of illness or with practical problems.

### 2.3. Statistical Analysis

Chi-square tests were used to describe the differences between the sample and the excluded persons. Univariate and multivariate binary logistic regression analyses were performed to examine the associations with internet use (dependent variable) and sex, age, education, household economy, living arrangement and cognitive functioning, health state, loneliness, and practical support (independent variables). For visualization purposes, rather than including interaction effects between age and other independent variables, multivariate binary logistic regression models were made stratifying on three age categories (65–74 years, 75–84 years, and 85+ years) as well as one model for participants of at least 75 years old only. The logistic regression models’ results are presented with average marginal effects (along with 95% confidence intervals) instead of odds ratios to ease interpretability. The *p*-values are calculated from Wald tests.

All statistical analyses were performed in SPSS [23] and/or R [24] using packages margins [25] and pscl [26].

## 3. Results

Comparing the included persons in the study to the nonrespondents, the included had significantly higher education (*p*-value = 0.029), better economic situation (*p*-value = <0.0001), and higher cognitive functioning (*p*-value = 0.005). No gender or age differences were found, although the tendency was that the included persons were younger (*p*-value = 0.068). Table 2 describes the characteristics of the sample, divided into internet users and non-users, and the excluded persons.

Among the study sample, 62.4% of the participants were internet users. This fraction strongly decreased with age: in the age group 65–74 years 87.1% were users, in the age group 75–84 years 60.3% were users, and in the age group of at least 85 years old 20.3% were users. Overall, the usage of the internet was more common among men compared to women. The study sample’s complete age and gender distributions are shown in Table 3, showing that women in the study are of older age.

In a descriptive analysis (Table 4), the non-users of the internet were categorized into groups of never users, persons who used the internet before, and persons who sometimes get help from others to use the internet. Reasons for being a non-user were most commonly that the persons did not know how to use the internet, had no need/interest in using the internet, or for other reasons not specified in the questionnaire. Less common reasons were that there was no possibility for broadband, the person was too busy, that the internet subscription or equipment was too expensive, or due to integrity or security reasons.

In the univariate logistic regression, factors associated with internet usage were lower age, male, higher education, good household economy, living with someone, having normal cognitive functioning, having a good state of health, and not being lonely (see Table 5). The only nonsignificant (at a 5% significance level) variable was practical support and was therefore not included in the multivariate models (Table 5 and Table 6). Loneliness was correlated with the living arrangement (correlation = 0.50), and to reduce any problem with multicollinearity, loneliness was not included in the multivariate models (living arrangement had the largest effect size and was therefore decided to be used over loneliness). The (McFadden) pseudo-R^2^-values of the multivariate models were 0.36 (Table 5), 0.22, 0.20, and 0.37 (models ordered by increasing age in Table 6).

The effect size of age in the univariate logistic regression decreased slightly in the multivariate logistic regression; for each year older a person gets, excluding effects of calendar time, his or her probability of being an internet user decreases by almost two percentage points (*p*-value < 0.0001, see Table 5). In age-stratified analyses, the effect was, however, only significant in the age group 75–84-year-olds (*p*-value = 0.002, see Table 6), but with a similar effect size in the oldest group, likely not significant (*p*-value = 0.120) due to small sample size (*n* = 118). In a model including all persons of at least 75 years old, the effect size of the decrease was 2.3 percentage points (*p*-value < 0.0001). Thus, there is evidence for age being strongly associated with non-use of the internet, where there is a decline in usage from 75 years. However, the exact age of 75 years is influenced by our choice of age-categories and should be thought of as approximate.

The univariate logistic regression (Table 5) stated that there was a 14.3-percentage-point-higher probability for men to be internet users than women. However, most of the difference is due to the women in the study being older—gender was not significantly associated with the use of the internet in the multivariate logistic regression model (Table 5). However, in the oldest age group, there was an adjusted 20.2-percentage-point-higher probability for men to be internet users than women (*p*-value = 0.014, Table 6).

In the non-stratified multivariate logistic regression model (Table 5), many of the other mentioned associations in the univariate logistic regression models had diminished. Younger age, higher education, better household economy, and higher cognitive functioning were still associated with being an internet user.

In the stratified multivariate logistic regression model (Table 6), apart from differing gender and residual age effects, there are some other differences across age categories. Education cannot be fully compared across age groups as the youngest, middle, and high education were grouped into one category (almost everyone with high education was internet users). In the oldest age group, the covariate household economy was excluded since not one person with poor economy was an internet user. Although not possible to measure, this covariate would clearly have had a strong association with internet usage. The exclusion of this covariate is also likely to explain the varying effects of cognitive functioning on internet usage, seen in Table 6. The oldest age group would have a 33.7-percentage-points-increase in the probability of being an internet user if the person had normal compared to poor cognitive functioning (*p*-value = 0.002), compared to an effect size of 14.7 percentage points in the non-stratified model (Table 5). In the oldest age group, there was a significant (*p*-value = 0.033) correlation between cognitive functioning and household economy, such that a better household economy was more common among persons with normal cognitive functioning.

## 4. Discussion

This study gives several interesting insights into older persons’ use of the internet in Sweden.

In Sweden, people started using the internet early. It also has one of the highest percentages of older adult internet users with a high digital maturity level, making this study interesting as a reference point for other countries. One significant result of this study is that research and demographic surveys most likely overestimate the use of the internet within the older population.

Our study finds that 62.4% of individuals over 65 years of age are internet users. Since we also have data on the nonresponders to this questionnaire, we also estimate that this is a roof value since the nonresponders display characteristics generally attributed to non-use, such as lower education, lower household economy, and lower cognitive functioning. It is likely that, despite lower proportions of internet users than other reports, our result still underestimates proportions between internet users and non-users.

In the large yearly survey on the internet habits of the Swedish population, “The Swedes and the Internet 2019 “(Svenskarna och Internet 2019 (SoI) [4]), the corresponding number is 84% for individuals over 66 years. SoI is the Swedish part of the World Internet Project, an international research project connected to the World Internet Institute that follows the spread and use of the internet worldwide.

SoI includes a comparable number of individuals to our study: 672 persons aged 66 and above (630 persons aged 65 and above in our study). The differences in the outcome can most likely be attributed to methodological issues. SoI is designed as a revolving panel design. This means that the base consists of a panel of people who are interviewed year after year. In the 2019 study, 57% participated in the survey via the web and 43% by telephone. The overall response rate was 16%.

It could be argued that the internet use of the group aged 65+ has a decreasing possibility to be correctly reported with this methodology and that the problems increase with age. As a comparison, the SoI reports an internet use of 93% in the age group 66–75, comparable to our study, where our study reports 87% in the age group 65–74. In the group 76+, the SoI reports 69% while in our study 75+ is reported as 49% with a substantial age gradient (75–84, 60% internet users and 85+, 20% internet users)

In another recent Swedish study [27] that also uses telephone interviews with a sample of the population, it was found that 80% of older adults have access to ICT, and the number of devices used decreases with age. In this study, the age group was limited to 65–85 years old, leaving the oldest age cohort excluded from examination.

That the younger-older adults use more internet than the oldest is a well-known fact and has been shown in other studies [3], it can also be seen that the older adults that have not been using the internet during their work-life are less likely to use the internet these days [6,28].

There seems to be a systematic underreporting of the internet use of oldest-old people, which could significantly impact how we perceive the digital divide and the possibility of digital health and welfare solutions for the older population. There is undoubtedly a strong cohort effect, and it could be argued that this large difference in use between the younger-older and the older-older will decrease with time. However, how strong this cohort effect is, we know little of at the moment. Since our study and others showed that cognitive abilities are an essential factor in internet use, there is today no way of saying how normal aging will affect internet use, especially for more frail groups. Neither how the constant development of new technologies and services can be adopted in, or adapted to, older age. What one was able to do on the internet, perhaps, is no longer possible due to a decline in motor skills, eyesight, or cognitive abilities. With the global demographic changes to an aging population, the prevalence of cognitive decline will increase, thus impacting older adults in society. To reduce the digital divide with the exclusion of the oldest old, the design and development of digital equipment and applications must consider this group’s needs. Properly managed internet use might contribute to a maintained cognitive function, high up in age, as shown in a study by Berner et al. [14].

As shown in this study, gender was linked to internet use. This agrees with previous studies where gender has also been associated with internet use; males are more likely to use the internet than females [5,29]. However, Hunsaker and Hargittai [3] suggested in their review that gender differences are not evident in every age cohort among older persons. As always, the older adult population cannot be treated as a homogeneous group, as significant cohort effects are present. According to the study of König et al. [5], sex is a significant predictor of internet use in age groups 66–79 and 85+ (not in 50–65-year-olds). Similar findings were also present in our study: in the oldest age group (85+), there was a 20.2-percentage-point-higher probability for men to be users than women, in contrast to no significant differences in the younger age groups. That is, in Sweden, gender differences in internet use will soon be evened out. Differences between studies are probably explained by how age, cohort, education, and working life are equated in the study population.

Our study has certain limitations. The study area represents two levels of urbanization, rural areas, and mid-sized cities. Large cities are not represented in the sample. However, when comparing our results in age cohorts where other Swedish studies have corresponding results, internet-use numbers are very similar, indicating a relatively high degree of generalizability [1,4].

## 5. Conclusions

This cohort study showed that 62.4% of the study subjects are internet users, but this could be slightly overestimated, as variables such as higher education, better economic situation, and higher cognitive functioning are all associated with higher internet use. However, higher internet use is still a digital divide among older adults which is why these variables should be taken into account to avoid digital exclusion. Thus, longitudinal and cohort studies to see how the cohort effects change over time are of importance.

Another conclusion is that when looking at the cohorts, it was evident that the gender imbalance between internet users and non-users that has been shown in many previous studies can be attributed to a cohort effect with a high imbalance among the oldest-old and almost no imbalance in the younger-old group.

## Figures and Tables

**Table 1:** Questionnaire dropout—the three first groups are included in the study sample.

	Frequency	Percent	Cumulative Percent
Respondent	581	79.3	79.3
* Actively declined to answer questionnaire	10	1.4	80.6
Dementia, other illnesses, accommodation, etc.	39	5.3	85.9
Nonrespondent to the questionnaire	103	14.1	100.0
Total	733	100	100

* A relative or other person sending back or communicating via telephone, that the person who received the questionnaire is unable to fill it out due to cognitive or physical reasons and that this also leads to non-use of the internet.

**Table 2:** Characteristics of the sample (*n* = 630) and the excluded persons (*n* = 103).

		Total Sample, *n* (%)	Not Internet User, *n* (%)	Internet User, *n* (%)	Non-Respondent, *n* (%)	Chi-Square Test: Sample vs. Non-Respondents (*p*-Value)
Total		630	237	393	103	
Gender	female	339 (54)	150 (63)	189 (48)	63 (61)	0.199
	male	291 (46)	87 (37)	204 (52)	40 (39)	
Age	65-74	225 (36)	29 (12)	196 (50)	24 (23)	0.068
	75-84	287 (46)	114 (48)	173 (44)	48 (47)	
	85+	118 (19)	94 (40)	24 (6)	31 (30)	
Education	low	267 (42)	160 (68)	107 (27)	56 (54)	0.029
	middle	185 (29)	44 (19)	141 (36)	24 (23)	
	high	142 (23)	17 (7)	125 (32)	12 (12)	
	NA	36 (6)	16 (7)	20 (5)	11 (11)	
Household economy	poor	41 (7)	28 (12)	13 (3)	15 (15)	<0.0001
	good	516 (82)	175 (74)	341 (87)	43 (42)	
	NA	73 (12)	34 (14)	39 (10)	45 (44)	
Living arrangement	alone	208 (33)	96 (41)	112 (28)	0 (0)	n.a.
	with someone	371 (59)	90 (38)	281 (72)	0 (0)	
	NA	51 (8)	51 (22)	0 (0)	103 (100)	
Cognitive functioning	poor	132 (21)	96 (41)	36 (9)	20 (19)	0.005
	normal	424 (67)	108 (46)	316 (80)	29 (28)	
	NA	74 (12)	33 (14)	41 (10)	54 (52)	
Health state	poor	196 (31)	90 (38)	106 (27)	0 (0)	n.a.
	good	376 (60)	94 (40)	282 (72)	0 (0)	
	NA	58 (9)	53 (22)	5 (1)	103 (100)	
Loneliness	no	415 (66)	113 (48)	302 (77)	0 (0)	n.a.
	yes	160 (25)	71 (30)	89 (23)	0 (0)	
	NA	55 (9)	53 (22)	2 (1)	103 (100)	
Practical support	no	26 (4)	6 (2)	20 (5)	0 (0)	n.a.
	yes	548 (87)	175 (74)	373 (95)	0 (0)	
	NA	56 (9)	56 (24)	0 (0)	103 (100)	

**Table 3:** Age and gender distributions among the users and non-users.

Age Category		Not Internet User, *n* (%)	Internet User, *n* (%)	Total
65–74	Female	15 (13)	97 (87)	112
	Male	14 (12)	99 (88)	113
	Total	29 (13)	196 (87)	225
75–84	Female	67 (44)	84 (56)	151
	Male	47 (35)	89 (65)	136
	Total	114 (40)	173 (60)	287
85+	Female	68 (89)	8 (11)	76
	Male	26 (62)	16 (38)	42
	Total	94 (80)	24 (20)	118
All (65+)	Female	150 (44)	189 (56)	339
	Male	87 (30)	204 (70)	291
	Total	237 (38)	393 (62)	630

**Table 4:** Type of non-user and reason for not using the internet among non-users (more than one reason per person is possible).

Type of Non-User	*n* (%)	
All	237 (100)	
Never user	55 (23)	
Used before	5 (2)	
Use the internet with someone else	113 (48)	
NA	64 (27)	
Reason for not using the internet	*n* (%)	Number of missing answers
No need/interest in it	109 (76)	93
Equipment is too expensive	32 (24)	103
Internet subscriptions too expensive	29 (22)	106
Do not know how to use the internet	109 (77)	95
For security or integrity reasons	37 (29)	108
No possibility for broadband where I live	7 (5)	105
No time/too busy	13 (10)	110
Other reasons	62 (50)	112

**Table 5:** Univariate and multivariate binary logistic regression analyses. Internet use as a dependent variable. Average marginal effects (AME) with 95% confidence intervals (CI) for the factors and *p*-values for the beta-coefficients.

		AME	CI	*p*-Value	Pseudo-R^2^	AME	CI	*p*-Value
Age		−0.028	(−0.030, −0.025)	<0.0001	0.21	−0.019	(−0.024, −0.015)	<0.0001
Sex	Female	Ref			0.02	Ref		
	Male	0.143	(0.070, 0.215)	0.0002		0.048	(−0.019, 0.116)	0.163
Education	Low	Ref			0.15	Ref		
	Middle	0.297	(0.234, 0.361)	<0.0001		0.166	(0.097, 0.235)	<0.0001
	High	0.455	(0.371, 0.538)	<0.0001		0.293	(0.206, 0.381)	<0.0001
Household economy	Poor	Ref			0.03	Ref		
	Good	0.321	(0.177, 0.464)	<0.0001		0.186	(0.047, 0.325)	0.010
Living arrangement	Living with someone	Ref			0.04	Ref		
	Living alone	−0.204	(−0.272, −0.136)	<0.0001		0.013	(−0.061, 0.087)	0.729
Cognitive functioning, MMSE	Poor	Ref			0.13	Ref		
	Normal	0.394	(0.337, 0.452)	<0.0001		0.147	(0.069, 0.225)	0.0004
Health state	Poor	Ref			0.04	Ref		
	Good	0.195	(0.125, 0.265)	<0.0001		0.029	(−0.038, 0.097)	0.393
Loneliness	No	Ref			0.02			
	Yes	−0.160	(−0.237, −0.084)	<0.0001				
Practical support	No	Ref			0.00			
	Yes	−0.096	(−0.296, 0.104)	0.346				

**Table 6:** Multivariate binary logistic regression analyses in three age categories. Internet use as the dependent variable. Average marginal effects (AME) with 95% confidence intervals (CI) for the factors and *p*-values for the beta-coefficients.

Age Category		65–74			75–84			85+		
		AME	CI	*p*-Value	AME	CI	*p*-Value	AME	CI	*p*-Value
Age		−0.002	(−0.019, 0.016)	0.854	−0.028	(−0.045, −0.012)	0.002	−0.020	(−0.044, 0.004)	0.120
Sex	Female	Ref			Ref			Ref		
	Male	0.012	(−0.068, 0.091)	0.777	0.008	(−0.110, 0.126)	0.900	0.202	(0.068, 0.335)	0.014
Education	Low	Ref			Ref			Ref		
	Middle	0.124 (Middle or High)	(0.037, 0.211)	0.004	0.263	(0.153, 0.373)	< 0.001	0.076	(−0.124, 0.275)	0.464
	High	−	−	−	0.367	(0.220, 0.513)	< 0.001	0.242	(0.064, 0.420)	0.023
Household economy	Poor	Ref			Ref			−		
	Good	0.096	(−0.024, 0.215)	0.127	0.161	(−0.096, 0.418)	0.224	−		
Living arrangement	Living with someone	Ref			Ref			Ref		
	Living alone	0.088	(−0.059, 0.234)	0.240	−0.024	(−0.145, 0.097)	0.693	−0.009	(−0.177, 0.159)	0.916
Cognitive functioning, MMSE	Poor	Ref			Ref			Ref		
	Normal	0.088	(0.002, 0.173)	0.046	0.107	(−0.047, 0.261)	0.179	0.337	(0.170, 0.504)	0.002
Health state	Poor	Ref			Ref			Ref		
	Good	0.039	(−0.050, 0.127)	0.394	0.056	(−0.056, 0.169)	0.330	0.008	(−0.153, 0.169)	0.924
